# Evidence-based design in an intensive care unit: End-user perceptions

**DOI:** 10.1186/s12871-015-0038-4

**Published:** 2015-04-25

**Authors:** Mauricio Ferri, David A Zygun, Alexandra Harrison, Henry T Stelfox

**Affiliations:** 1 Department of Community Health Sciences, Institute for Public Health, University of Calgary, Calgary, Canada; 2 Division of Critical Care, University of Alberta, and Alberta Health Services, Edmonton, Canada; 3 Department of Critical Care Medicine, University of Calgary, and Alberta Health Services, Calgary, Canada; 4 Department of Medicine, University of Calgary, and Alberta Health Services, Calgary, Canada

**Keywords:** Critical care, Facility design and construction, Health care evaluation mechanisms, Qualitative research, Post-occupancy evaluation

## Abstract

**Background:**

The objective of this study was to describe end-user impressions and experiences in a new intensive care unit built using evidence-based design.

**Methods:**

This qualitative study was comprised of early (2–3 months after opening) and late (12–15 months after opening) phase individual interviews with end-users (healthcare providers, support staff, and patient family members) of the newly constructed Foothills Medical Centre intensive care unit in Calgary, Canada. The study unit was the recipient of the Society of Critical Care Medicine Design Citation award in 2012.

**Results:**

We conducted interviews with thirty-nine ICU end-users, twenty-four in the early phase and fifteen in the late phase. We identified four themes (eleven sub-themes): atmosphere (abundant natural light and low noise levels), physical spaces (single occupancy rooms, rooms clustered into clinical pods, medication rooms, and tradeoffs of larger spaces), family participation in care (family support areas and social networks), and equipment (usability, storage, and providers connectivity). Abundant natural light was the design feature most frequently associated with a pleasant atmosphere. Participants emphasized the tradeoffs of size and space, and reported that the benefits of additional space (e.g., fewer interruptions due to less noise) out-weighed the disadvantages (e.g., greater distances between patients, families and providers). End-users advised that local patient care policies (e.g., number of visitors allowed at a time) and staffing needed to be updated to reflect the characteristics of the new facility design.

**Conclusions:**

End-users identified design elements for creating a pleasant atmosphere, attention to the tradeoffs of space and size, designing family support areas to encourage family participation in care, and updating patient care policies and staffing to reflect the new physical space as important aspects to consider when building intensive care units. Evidence-based design may optimize ICU structure for patients, patient families and providers.

**Electronic supplementary material:**

The online version of this article (doi:10.1186/s12871-015-0038-4) contains supplementary material, which is available to authorized users.

## Background

Expanding health services and design-focused research in conjunction with a growing emphasis on quality improvement in healthcare systems has contributed to the emergence of evidence-based design [1-3]. Evidence–based design is defined as the application of the best available knowledge from research and practice to take design decisions (i.e., healthcare physical environment) [3]. This novel approach extends beyond minimum construction standards and aims to recommend healthcare facility design features that improve clinical performance and create “healing environments” [1,3,4].

Evidence-based design may be particularly relevant for intensive care units (ICU) in which patients have life-threatening conditions and the model of care is based on multidisciplinary teamwork. Intensive care units leaders have been challenged to increase quality, reliability, and safety of service delivery in recent years [5-7]. Facility design affects the social behavior of end-users. It potentially shapes the way patients, families, and providers interact and impacts processes of care and patient outcomes [1,6,8,9].

The objective of this study was to describe end-user impressions and experiences in a new ICU constructed using evidence-based design [10]. The setting for our study was the new Foothills Medical Centre intensive care unit (FMC-ICU) in Calgary, Canada, recipient of the Society of Critical Care Medicine (SCCM) ICU Design Citation award in 2012 [11]. Clinicians and design experts collaborated in the planning process that started with defining their vision of the ideal facility and developing guidelines which incorporated state-of-the-art technology and functionality in a pleasant environment for end-users. Subsequently, the planning team used life-size simulation to determine room configuration and equipment disposition [12]. The new FMC-ICU incorporated design features such as single-occupancy rooms, patient care rooms clustered into clinical pods, and dedicated family support areas [1,3]. We took advantage of the opening of the FMC-ICU to determine end-user impressions and experiences with these evidence-based design features.

## Methods

### Approach

This was a qualitative study comprised of interviews with the end-users of the FMC-ICU. We conducted two phases of data collection to account for both the “settling–in” period, when problems are most frequent, and the “halo effect”, associated with moving to a new facility [4]. Early phase interviews were conducted two to three months after the facility opened and late phase interviews were conducted twelve to fifteen months after it opened. We obtained verbal informed consent from all study participants. The Conjoint Health Research Ethics Board at the University of Calgary approved the study protocol (E-24609).

We generated an interview guide using a focused literature review, examination of local guidelines for ICU design, and interviews with four key informants who served on the local design committee. The interview guide (see Additional file 1) consisted of open-ended questions to encourage participants to freely reflect on their impressions and experiences with the new FMC-ICU as well as probing questions regarding specific evidence-based design features.

We utilized a non-probability sampling strategy (maximum variation purpose sampling) to obtain a wide range of perspectives representing the typical diversity of end-users’ groups experiencing the new ICU facility. We included healthcare providers, support staff and patient family members. The study was locally publicized with electronic messages to inform providers of the purpose, time, and location of the interviews. End-users were recruited during typical working shifts to facilitate participation.

### Analysis

We analyzed verbatim transcripts of individual, semi-structured, in-depth audiotaped interviews. We used traditional qualitative analysis with an iterative and reflexive process [13,14]. This involved multiple reviews of the transcripts, identifying key concepts as codes, using constant comparison to refine and modify the codes, grouping broad topics into themes through careful reading and re-reading of the data. One author (MF) coded and analyzed six transcripts and concomitantly developed a qualitative codebook to define, code, and synthetize the core ideas expressed by the participants. Subsequently, we divided themes into sub-themes that described the findings in more detail. All authors reviewed and revised the coding scheme which one author (MF) subsequently applied to all transcripts with minor adjustments in the following iterations.

As a final step in the data analysis, we developed a theoretical framework to classify and visualize end-user perceptions of structure, process, and outcomes across the ICU functional zones (i.e., physical areas housing a set of interrelated functions) [1]. We merged the Donabedian conceptual model for quality of healthcare delivery and the 2012 SCCM Guidelines for Intensive Care Unit Design to inform our approach [1,15,16].

## Results

We interviewed thirty-nine end-users of the FMC-ICU, twenty-four in the early phase and fifteen in the late phase. Table 1 describes participants’ characteristics grouped by study phase. Our analysis produced four themes (atmosphere, physical spaces, family participation in care, and equipment) and eleven sub-themes (Table 2).

**Table 1:** **Participant characteristics**

	Early phase	Late phase
Age, years, median (IQR)	36 (31–48)	38 (32–49)
Female (%)	54	60
Work experience^†^, years, median (IQR)	8.5 (3–18)	10 (4–15)
Number of participants	24	15
Nurse	8	5
Respiratory therapist	4	3
Physician	3	3
Other providers	5	0
Support staff	3	1
Family member	1	3

Participant group identified as “Other providers” includes physiotherapists, social workers, and dietitians. “Support Staff” group includes unit clerks and cleaning staff members. † “Work experience” refers exclusively to healthcare providers.

**Table 2:** **Themes and sub-themes identified from end-user interviews**

Themes	Sub-themes	Comments
**Atmosphere**		
	*Abundant natural light*	Bright rooms with ample windows providing natural light and views of nature are calming and boost mood/morale for families and providers (n = 105).
	*Low noise levels*	Quiet environment improves concentration, task completion, and teamwork (n = 40). Sign of respect for patients.
**Physical spaces**		
	*Single-occupancy rooms*	Positive aspects (n = 69, e.g., privacy, family presence at bedside). Negative aspects (n = 30, e.g., safety concerns given increased distance between patients and providers).
	*Rooms clustered into clinical pods*	Positive aspects (n = 4, e.g., ICU seems less busy). Negative aspects (n = 75, e.g., less situational awareness).
	*Medication rooms*	Positive aspects: Large room for multiple users at peak time (n = 9). Quieter with less distraction during preparation (n = 2). Negative aspects: nurses can’t hear bedside alarms (n = 4). Need for extra staff coverage (n = 2).
	*Tradeoffs of larger spaces*	Positive aspects (n = 56) of larger spaces such as facilitated teamwork activities (e.g., rounds without interruption) are worth the negative aspects including patient safety concerns. Additional measures are necessary to mitigate some negative aspects (n = 8).
**Family participation in care**		
	*Family support areas*	More space in family areas is functional (n = 17), with location (n = 19) and flexibility (n = 2) important.
	*Social networks*	Location and configuration impact informal networks with other families (n = 2). Connectivity for family members (n = 2).
**Equipment**		
	*Usability*	Positive: Innovative equipment (n = 9). Negative: Challenges using new equipment in early phase (n = 8).
	*Storage*	Positive: Same storage configuration in all clinical pods (n = 8). Supplies in the room (n = 2).
	*Provider connectivity*	Positive: More computers to access and document clinical information (n = 19)

Numbers in parentheses represent the total number of comments about a sub-theme.

### Atmosphere

All participants mentioned a brand new ICU with a pleasant atmosphere as a major positive impact in both phases. The effects of a pleasant atmosphere were apparent for patient families and providers, who both reported that they resulted in calmer families more willing to interact with the provider team. The most impactful elements of atmosphere were natural light and lower noise levels.

*“Well, number one, it’s brand new (…) So that’s very impressive. The room that my husband is in is wonderful (…) size-wise, beautiful view.”*

#### Abundant natural light

It was the most frequent component of a pleasant atmosphere described. The majority of participants highlighted the perceived benefits of a brighter facility with more windows. Three noted that the ICU was bright during the day and adequately dark during the night, creating conditions for day/night cycle. The related construct “views of nature” was identified as having a positive impact on the mood and morale of end-users.

*“It’s bright, because so much of our work is dark. You know, it’s heavy, it’s emotion-laden, it’s fearful, it’s stressful, there’s a lot of death, there’s loss. You know, it’s depressing. So it is really important to have a bright, colourful environment”*

*“At night it’s darker I think, whereas before we had [artificial] skylights and … it wasn’t always as dark.*”

#### Lower noise levels

Participants associated lower noise levels with better concentration and ability to complete clinical tasks, such as rounding, with fewer interruptions (n = 20). They perceived lower noise levels were a sign of respect to patients and family members. As a negative point, the unit was perceived as noisy during handover between work shifts. Providers suggested the cause was the bedside design with decentralized nursing stations and two teams of nurses sharing the same workspace.

*“The most significant change that I have seen is the fact that the unit is very quiet. You can round in an ambiance that allows you to concentrate on -- on your work. You no longer have the multiple distractions that we used to have in the -- in the previous unit (…) It was so noisy and so very many interruptions that oftentimes I was concerned with my ability to -- to maintain my -- my concentration”*

### Physical spaces

End-users identified three physical spaces as important: single-occupancy rooms, rooms clustered into clinical pods, and medication rooms. Many end-users made both positive and negative comments about overall unit size and space. Some identified tradeoffs of size and space, including the challenge of larger physical spaces resulting in greater distances between patients, patient families and providers.

#### Single-occupancy rooms

Sixty-nine comments described positive aspects of single-occupancy rooms: privacy for patients and families (n = 20), confidentiality during clinical encounters (n = 25), room to accommodate providers during routine and emergency care, and presence of family members at the bedside (n = 18). Six participants indicated that single-occupancy rooms allowed for better infection prevention and control practices. Three family members commented that the care provided in single-occupancy rooms was perceived as more individualized.

*“There’s just the space to move, there’s space to get equipment in and out, there’s space to get people in and out. It just makes it easier to actually do your job ‘cause there’s physically the space to do it”*

In the early phase, participants mentioned safety concerns, including increased distance from patients, lack of visual contact, and perceived difficulty hearing alarms (n = 12), feeling isolated from other providers (n = 8), and concerns about calling for help from inside the room (n = 5). In the late phase, nurses did not mention isolation but still commented on concerns about calling for help from inside the room (n = 3) and the distance from the patients (n = 2).

*“I would say the four walls around us kind of hinders maybe sometimes getting help. Like there used to be curtains between us, and you could see the feet shuffling underneath and you’d say hey you, you know, I need help. Now it’s not quite as easy to just call out for help”*

*“… before you could just open the curtains, you could see six patients, so you could help whoever was crashing, here you can’t because you just can’t see more than two patients at a time.”*

#### Rooms clustered into clinical pods

There were seventy-five comments on negative aspects of patient rooms clustered into clinical pods. The comments suggested that the clinical pods were excessively spread-out, which hindered social interaction and camaraderie among providers (n = 11) and reduced visual contact between providers, “hampering” situational awareness (n = 20). Although participants thought it was more difficult to find people within the unit, this was not perceived to impact informal professional support networks (e.g., asking someone else for clinical advice) (n = 9). Nine participants indicated the layout was a barrier to diverting providers to busier areas within the ICU when help was needed. Physicians noted increased walking distances made it more physically demanding to look after patients in different pods. Nurses commented that cross coverage of patients was more difficult with larger physical spaces, and that this challenge had not been anticipated prior to opening the new ICU (n = 6).

“*Again, the physical plant is so spread out; when we have to cover you don’t know what’s going on in the units (clinical pods). And for us, that’s tough when you’re on call, right? Like if somebody’s really sick, they have to be able to get a hold of you, you have to get there. So that’s one thing.”*

*“We used to always be so close together that we were all tight in the same place, which led to a more social atmosphere, I found. Sometimes you feel—I don’t want to say isolated, but sometimes you feel like everyone’s very far away and there’s not that camaraderie and immediately social aspect.”*

There was no mention of any positive aspects of rooms clustered into clinical pods in the early phase. In the late phase, four participants mentioned that this arrangement facilitated identification of the ICU team by family members and consultants. They also commented that separate clinical pods allowed end-users to avoid exposure to events occurring in other areas of the ICU (e.g., noise and activity associated with movement of patients in and out of the ICU or stress of patient resuscitation).

*“…I like the separate pod idea. Because if you were in the open unit, when it was busy in one side, it felt like the whole unit was busy, right? And it would be just wearing on you.”*

#### Medication rooms

Nurses mentioned the impact of a dedicated and distinct medication room twenty times. The size allowed multiple providers to use it simultaneously at peak medication hours (n = 9). Lower noise levels were reported to lead to perceptions of less distraction during medication preparation, fewer errors and increased safety (n = 2). The main negative point was the need for nurses to have their patients monitored by a colleague while using the medication room because they could not hear alarms from the room (n = 4).

*“I guess it’s nice that it’s divided up—three, one in each pod. It’s not a mad rush and first thing in the morning, you’re not fighting. So I guess that’s something.”*

*“If I have to go and get a medication, it’s hard to hear your patient, right? If there’s an alarm or something, … and sometimes if you have a really sick patient, it’s a little bit sketchy running to get a med.”*

#### Tradeoffs of larger spaces

All participants indicated that the positive aspects of more space outweighed the negative. End-users suggested that more space facilitated the presence of family members at the bedside. They also felt that more contact with the provider team reassured families about the quality of care provided and improved the perception of coordinated teamwork. Providers commented that larger hallways and more space at the bedside facilitated seamless teamwork activities including fewer interruptions during multidisciplinary clinical rounds. Nurses mentioned that the implementation of a wireless provider-to-provider communication system (n = 5) was perceived as good solution for difficulties created by a larger unit. Providers felt subsequent improvements in the emergency response system within the ICU (i.e., providers could trigger a local code call from inside the room with the touch of one button) increased safety in a larger space (n = 3). In addition, end-users commented that local policy and guidelines were outdated and limited the perception of improvement brought by larger spaces (e.g., policies restricting the number of visitors at the bedside to two).

### Family participation in care

#### Family support areas

End-users identified the location of family areas and other hospital amenities (n = 19). Close but physically separated from the patient care area were identified as being important to end-users. Participants highlighted positive (n = 6) and negative (n = 3) aspects of family support areas size. They suggested larger areas were important to accommodate larger groups of visitors in a comfortable way. They indicated they helped a diverse group of visitors to simultaneously use the space. In contrast, one provider mentioned that large areas felt cold and impersonal. Conversely, smaller rooms were more intimate, but visitors “camped” in them, limiting access for others.

*“So it would be nice if we had, you know, a big room, a big waiting room with some small rooms that could be used for privacy for some people, right, within that big unit, that big… big waiting room. So it seems a bit cold in there. “*

*“But I actually think that they are very much appreciated by families and you’re making a statement, which is (…) you are thinking about not just the patients, not just the staff, but the families that often have a 24-hour presence as long as people are in the ICU”*

#### Social networks

End-users suggested that the family support area location was crucial to developing informal social networks among visitors facilitating way-finding and transitions of care out of the ICU. A healthcare provider questioned if more flexible rooms, with smaller and more private areas, could enhance interactions between families of different patients, facilitate sharing of experiences, and help create family networks. Two family members appreciated the availability of computers with internet access and free public telephones, which enabled communication with relatives and friends.

*“And it helps to talk to people, I think (…) Most people want to talk about their situation, right?“*

### Equipment

#### Usability

Participants commented on equipment usability seventeen times. In the early phase, providers identified negative aspects of new equipment usability such as problems getting used to the dual pendant-mounted system, with medical gases and power outlets suspended from the ceiling (n = 4), and malfunctioning automatic doors, impeding rapid access between units (n = 4). However, in the late phase, they indicated that having innovative equipment (e.g., ability to display patient monitor information on the room television screen) was perceived as positive and helpful to patient care (n = 9).

*“The arms are very frustrating because before you could decide where your ventilator was, where your IV pumps and if you did a bronch, I would pull my IV pumps to the foot of the bed so it would be out of everyone’s way.”*

#### Storage

Participants indicated that the storage of supplies and equipment needed to be identical in each clinical pod so that providers knew where to find them (n = 5). Two nurses indicated that the presence of supplies within patient rooms facilitated workflow.

#### Provider connectivity

Providers perceived increased computer availability as a positive feature to access information and document clinical care (n = 19). In contrast, two physicians suggested it could be distracting from clinical tasks.

*“Also the increased availability of lots of computers. So you’re not fighting for space or a computer to do recording.”*

## Discussion

Our analysis provides an opportunity to understand how evidence-based design impacts the impressions and experiences of ICU end-users. Abundant natural light and low noise levels improved the perception of a pleasant atmosphere. Participants emphasized tradeoffs of size and space, identifying safety concerns of increased distance between end-users. Physical and functional characteristics of family support areas influenced the reported integration of family members in patient care. Unit polices needed adjustment to reflect the new facility design.

Despite a growing body of evidence, conflicting study results have not allowed conclusive identification of essential design features to transform ICUs into “healing environments” [8,17,18]. However, the SCCM ICU design guidelines recommended features that were identified as important in our study included natural light, low noise levels, single occupancy-rooms, unit arrangement with rooms clustered into clinical pods, and family support areas [1].

The guidelines suggested natural light is essential and recommended at least one window per patient bed area [1]. This is supported by the findings in this study since abundant natural light was the most frequently identified feature for a pleasant atmosphere. However, a recent secondary analysis of a large cohort study in patients with brain injury did not show improvement in patient outcomes with the presence of windows in ICU rooms [17].

End-users in this study described benefits of lower noise levels. In addition to facilitating patient sleep, which has been described elsewhere [19-22], they suggested it improved concentration and task completion (e.g., clinical rounds) and thought quietness was a sign of respect for the families. In agreement with previous studies, one provider commented that family members appeared calmer and more willing to engage with the team as a result of a quieter atmosphere [8,18].

The latest SCCM guidelines for the management of pain, agitation, and delirium in adult patients recommended promoting sleep through strategies to optimize the ICU atmosphere (i.e., light and noise level control). Accordingly, end-users perceived these two components of atmosphere as import for a more pleasant environment [23].

Single-occupancy rooms are standard in new North American ICUs [19]. They have been associated with lower infection transmission rates, more privacy, and improved satisfaction [20,24-26]. The perceptions of end-users in this study corroborated these findings. However, nurses identified safety concerns related to increased distance from the patients. Units with lower nurse-to-patient ratios may need to consider this important tradeoff [26].

The SCCM guidelines suggest considering unit arrangement with rooms clustered into clinical pods when the number of beds exceeds twelve [1]. A recent review of recipients of the SCCM Design Citation ward showed larger units with clinical pods as a rising trend [27]. In our study, there was little support for clustering rooms into separated clinical pods. End-users of FMC-ICU perceived this design feature to be associated with the negative aspects of an overall larger unit including decreased situational awareness and excessive walking. These observations suggest that there may be important tradeoffs between ICU size and organization of space and that opportunities exist to further improve ICU layout and room arrangement

Family areas are designed to satisfy a wide array of visitor needs [1-3]. Flexible room sizes and configurations facilitate accommodation and privacy [1-3,8,18]. Our findings concur with room flexibility as a main attribute enabling private interactions among end-users who wanted to exchange experiences while integrating diverse groups of visitors in the same space. The literature suggests that families rearrange furniture and seating if their needs are not met [1-3]. Social networking may also be impacted by room configuration and location. As patients were discharged to other hospital wards, families seemed to value proximity to the ICU to keep informal relationships and social networks built during the stay.

Another unique contribution of this study is the account of how unit policy and practice guidelines may influence the perceived impact of structural interventions on processes and outcomes of care. This finding highlights a, potentially, overlooked aspect of facility construction that could impede the realization of the full benefits of a new facility.

### Key Messages

Our interpretation of end-user perceptions of the impact of structural changes on experiences provides important insights that can inform the design of future ICUs (Table 3):

- End-users find intensive care unit atmosphere important, specifically, natural light and low noise levels.
- Larger spaces require attention to the tradeoffs of size. Larger spaces facilitate family presence and increase the perception of high quality of care. However, safety concerns about increased distance between providers and patients require careful consideration.
- Flexible space configurations, access to hospital amenities (e.g., vending machines, parking), and internet connectivity contribute to visitor satisfaction.
- Design features need to be supported by changes in unit policy, guidelines, and staffing.

**Table 3:** **Potential relationship between Design Features, Processes and Outcomes of care**

Design features (Structure)	Processes	Outcomes
**Patient Care Zone**		
1. Ample windows	Abundant natural light	Increased end-user satisfaction
	Access to views of nature	Potential for less patient anxiety and stress*
2. Adjustable light level	Improved day/night cycles	Increased end-user satisfaction
3. Noise control measures	Lower noise levels, improved teamwork, calmer visitors, improved visitor-provider interactions	Increased end-user satisfaction
	Fewer interruptions, improved provider concentration	Potential for improved task completion*
4. Single-occupancy rooms	Increased visitor presence at bedside, improved visitor-provider interactions	Improved end-user satisfaction, potential for improved confidentiality/privacy*
	Difficult to hear bedside alarms	Potential for more adverse events*
5. Large patient care area	Increased number of providers at bedside, improved teamwork, improved provider-provider interaction	Increased end-user satisfaction
	More walking, isolated providers, decreased provider-provider interaction	Decreased end-user satisfaction
6. Rooms clustered into clinical pods	Decreased provider situational awareness, fewer provider social interactions, more walking, increased number of providers required for coverage, decreased teamwork	Decreased end-user satisfaction, potential for more adverse events*
	Easier identification of caring team, reduced exposure to activities not related to patient care	Improved end-user satisfaction
7. Storage of supplies in the room	Increased access and utilization of supplies	Improved end-user satisfaction
8. More computers	Improved medical documentation	Improved end-user satisfaction
9. New equipment training	Improved early usability	Improved end-user satisfaction, potential for fewer adverse events*
10. Decentralized nursing stations	Higher noise levels	Decreased end-user satisfaction
**Clinical Support Zone**		
1. Restricted access to medication room	Fewer interruptions during medication preparation	Potential for fewer adverse events
	Difficult to hear bedside alarms	Potential for more adverse events*
2. Large medication room	Improved utilization by multiple providers at peak hours	Potential for fewer adverse events*, improved end-user satisfaction
**Unit Support Zone**		
1. Provider areas close to the ICU	Increased utilization by providers	Improved end-user satisfaction
2. Large provider support areas	Increased utilization by providers	Improved end-user satisfaction
3. Administrative offices close to the ICU	Increased provider-decision-maker interactions	Improved end-user satisfaction
4. Same storage configuration in all clinical pods	Improved access to and utilization of supplies	Improved end-user satisfaction
**Family Support Zone**		
1. Family area location close to areas of interest to visitors	Increased visitor presence, improved visitor-visitor interaction, easier wayfinding	Improved end-user satisfaction
2. Flexible family area configuration	Easier to accommodate diverse needs	Improved end-user satisfaction
3. Access to free internet and telephone	Improved communication, increased visitor presence	Improved end-user satisfaction

Framework developed merging the Donabedian conceptual model and the 2012 Society of Critical Care Medicine Guidelines for Intensive Care Unit Design (support zones). Design Features (STRUCTURE) are design elements perceived as important by study participants. PROCESSES of care are end-user activities while giving or receiving healthcare-related actions. OUTCOMES of care are the effects perceived by end-users. End-users may include healthcare providers, support staff, and family members. *Outcomes marked as potential given the exploratory nature of the relationships based on end-user perception.

### Limitations

Recall bias is a potential limitation of interviews. We conducted two sets of interviews following the relocation to attenuate this risk. The format, content, and order of the questions and probing points may have lead participants to comment on or omit specific aspects of their experience. We minimized this by generating an interview guide and probing points with open-ended neutral questions based on a rigorous process to identify design elements that would have the greatest impact participants’ experience. Selection bias is another risk with small number of participants. Overall, the sample represented the typical diversity of ICU end-users’ groups, and frequent visits to the ICU with ample publicity of the interviews ensured inclusion of participants from different shifts and teams. To mitigate potential personal biases and ensure that the coding and analysis reflected the voice of end-users, our review and audit of the coding process included an experienced qualitative researcher who did not work in the FMC-ICU. Finally, although our study focused on a single newly constructed ICU, we believe that end-user perceptions of evidence-based design features are relevant for other centers.

## Conclusion

End-users identified a pleasant atmosphere (natural light and low noise levels), attention to the tradeoffs of space and size, designing family support areas to encourage family participation in care, and updating patient care policies and staffing to reflect the characteristics of new physical space as important elements to consider when building intensive care units. Evidence-based design may be used to optimize ICU structure for patients, patient families, and providers.

## Additional file

Additional file 1: **Interview guiding questions and probing points.** †Question asked only to family members.

## Abbreviations

ICU: Intensive care unit
FMC-ICU: Foothills medical centre intensive care unit
SCCM: Society of critical care medicine
IQR: Inter quartile ranges
