# Supplementary material for: Evidence-based design in an intensive care unit: End-user perceptions
Source: BMC Anesthesiol. 2015 Apr 25;15:57. doi: 10.1186/s12871-015-0038-4 (PMC4414278; doi:10.1186/s12871-015-0038-4)
Supplement: Additional file 1: — Interview guiding questions and probing points. †Question asked only to family members. [file 12871_2015_38_MOESM1_ESM.docx]

Supplemental Digital Content 1. Interview guide and probing points.

| **Questions** | **Probing points** |
| --- | --- |
| 1. Positive and negative aspects of the new ICU design? | Atmosphere  Single rooms  Geographic location |
| 2. Is your job easier or more difficult because of the facility design? | Bedside technology  Access to supplies  Medication room/errors |
| 3. Could you describe a specific event when there was an emergency situation and the space facilitated/hindered your delivery of patient care? | Intra-unit code activation system  Cardiac arrest team/ Medical emergency team response time. |
| 4. In terms of workflow how would you describe the new FMC-ICU facility? |  |
| 5. In terms of communication how does the FMC-ICU fare? |  |
| 6. Besides your job, how does the facility affect your work team, interaction with other care providers, patients and family?  † How does the facility affect your interaction with care providers, your loved-one, and other families? | Social networks.  Providers support areas.  Family support areas. |
| 7. If you could fix one thing about the new ICU what would that be? |  |
| 8. Which aspects of the new ICU do you like the most? | Layout, single rooms, atmosphere, family areas, or any other aspect mentioned by the respondent. |
| 9. Is there anything else you would like to tell us about the new ICU building? |  |
